# Burden of Disease in Pediatric Tumor-Induced Osteomalacia: A Literature Review

**DOI:** 10.1007/s00223-026-01580-0

**Published:** 2026-08-10

**Authors:** Salvatore Minisola, Suzanne M. Jan de Beur, Thomas O. Carpenter, Kathryn M. Dahir, María Belén Zanchetta, Leanne Ward, Ben Johnson, Neil Webb, Cody Broadhurst, Victoria Ward, Erik A. Imel

**Affiliations:** 1 Department of Medical and Cardiovascular Sciences, “Sapienza” University of Rome, Rome, Italy; 2 Division of Endocrinology and Metabolism, Department of Medicine, University of Virginia, Charlottesville, VA USA; 3 Departments of Pediatrics (Endocrinology) and Orthopaedics and Rehabilitation, Yale School of Medicine, New Haven, CT USA; 4 Division of Endocrinology and Metabolism, Vanderbilt University Medical Center, Nashville, TN USA; 5 Instituto de Investigaciones Metabólicas, Universidad del Salvador, Buenos Aires, Argentina; 6 Department of Pediatrics, Division of Endocrinology and Metabolism, Children’s Hospital of Eastern Ontario, University of Ottawa, Ottawa, Canada; 7 Kyowa Kirin International, Marlow, UK; 8 Source Health Economics, 226 Banbury Rd, Summertown, Oxford, OX2 7BY UK; 9 Departments of Medicine and Pediatrics (Endocrinology), Indiana University School of Medicine, Indianapolis, IN USA

**Keywords:** Clinical burden, Pediatric, Tumor-induced osteomalacia, Literature review

## Abstract

Tumor-induced osteomalacia (TIO) is an ultra-rare, paraneoplastic syndrome caused by tumors secreting fibroblast growth factor 23 (FGF23). In children, TIO may be mistaken for more common causes of rickets and osteomalacia, including monogenic forms, leading to long diagnostic delays. This review aimed to identify evidence on the diagnostic journey and burden of TIO in pediatric patients. A literature review was conducted to identify publications reporting disease characteristics, investigations, treatments, and clinical outcomes in pediatric patients diagnosed with TIO. In total, 41 studies were included in the review, reporting on 46 pediatric patients. Mean age at presentation was 11.2 years (standard deviation [SD]: 4.6). The majority of individuals (60.9%) were male. The most commonly reported symptoms at presentation were pain (65.2%), weakness (47.8%), and impaired physical function (43.5%). Rickets was reported in 45.7% of patients and fractures in 34.8% of patients. Mean time from onset of symptoms to diagnosis was 4.3 years (SD: 2.6) and mean number of imaging procedures per patient was 5.4 (SD: 2.7). Low serum phosphate concentration for age was reported in 93.5% of patients at presentation. Attempted surgical resection was reported in the majority of patients (89.1%) and was successful in 60.9%. Pediatric-onset TIO is associated with a substantial symptomatic and healthcare burden. Increasing awareness of TIO in children may prevent delays in diagnosis, reduce the need for radiation-conferring tests, and lower morbidity due to effective management. Further research in this area is needed to address the scarcity of data available in pediatric patients.

**Supplementary Information:**

The online version contains supplementary material available at https://doi.org/10.1007/s00223-026-01580-0.

## Introduction

Tumor-induced osteomalacia (TIO) is an ultra-rare paraneoplastic syndrome caused by tumors secreting fibroblast growth factor 23 (FGF23) [1], a phosphate- and vitamin D-regulating hormone [2]. The resulting excess FGF23 decreases renal tubular reabsorption of phosphate (TRP) and impairs production of 1,25-dihydroxyvitamin D (1,25(OH)_2_D), resulting in chronic hypophosphatemia [3]. Patients with TIO present with musculoskeletal symptoms that may include fractures, pain, fatigue, and severe weakness [4, 5]. Pediatric patients with TIO may also exhibit features such as delayed motor milestones, difficulties ambulating, and features of rickets (including lower extremity deformity) [3, 6]. However, signs and symptoms are non-specific; therefore, TIO may remain insidious, leading to long delays in diagnosis. TIO may also be mistaken for more common causes of rickets and osteomalacia such as vitamin D deficiency rickets [7], or heritable forms of FGF23-mediated hypophosphatemia such as X-linked hypophosphatemia (XLH) [8], which also contributes to diagnostic delays.

Due to its rarity, data on the prevalence and incidence of TIO are scarce. An epidemiologic study of TIO in Denmark reported a total population incidence rate of 0.13 per 100,000 persons per year [9] while a German study reported a total population incidence rate of 0.094 cases per 100,000 persons per year and a prevalence of 0.187 cases per 100,000 persons [10]. Pediatric cases (aged < 18 years) of TIO are even more rare: a recent literature review of 610 published cases of TIO reported only 34 pediatric cases (5.6%) [11].

If the tumor can be located and is surgically accessible, complete surgical resection of the causative tumor is curative in most cases. However, incomplete resection of the tumor often results in persistence or recurrence of hypophosphatemia and associated symptoms [12]. Additionally, approximately 35–40% of patients with suspected TIO have tumors that cannot be localized [3, 13, 14]. Searching for a monogenic cause of FGF23-mediated hypophosphatemia is prudent prior to a comprehensive search for a tumor, to avoid unnecessary imaging [3]. In cases where the tumor cannot be localized or completely resected, treatment with oral phosphate salts and active vitamin D or burosumab is recommended [3, 15]. However, treatment with oral phosphate salts and active vitamin D is burdensome, as it must be administered 4–6 times a day, and is often not well tolerated, leading to poor adherence [15]. This is particularly burdensome in children, where arrangements for medications to be administered at school are needed, adults must oversee multiple daily doses, and gastrointestinal upset can cause soiling at school and home. Such treatment can also be associated with complications including nephrocalcinosis, nephrolithiasis, and secondary/tertiary hyperparathyroidism in both children and adults [12, 15]. Burosumab, a fully human monoclonal antibody that inhibits the activity of FGF23, has been approved in several regions worldwide for the treatment of TIO associated with tumors that cannot be curatively resected or localized [16, 17].

Evidence regarding TIO in pediatric patients remains limited; therefore, a literature review was conducted to identify and summarize the available published evidence that describes the diagnostic journey and burden of TIO in pediatric patients.

## Methods

A targeted literature review was conducted using electronic databases to identify publications reporting on disease characteristics, investigations, treatments, and clinical outcomes in pediatric patients diagnosed with TIO.

### Eligibility Criteria

The population, intervention, comparator(s), outcomes, and study design (PICOS) elements for the literature review are presented in Table 1.

**Table 1:** Eligibility criteria (PICOS)

Characteristics	Inclusion criteria	Exclusion criteria
Population	Pediatric patients (aged < 18 years at presentation) with TIO Studies with a mixture of adult and pediatric patients were included if subgroup data were reported for pediatric patients	Adults (aged ≥ 18 years at presentation) Patients without TIO
Intervention/ comparators	No restriction	–
Outcomes	Clinical, humanistic, and economic burden of disease and diagnostic odyssey associated with TIO, including but not limited to: Patient characteristics (e.g. age, gender, geography) Disease characteristics (e.g. tumor location, dimension, benign/metastatic) Clinical manifestations (e.g. fractures/pseudofractures [including frequency and location], rickets (including rachitic rosary), impaired linear growth, skeletal deformities and symptoms (e.g. pain, muscle weakness, fatigue, impaired physical function) Tumor localization procedures (e.g. type and frequency of imaging tests, biopsies) Orthopedic surgical procedures (e.g. fracture fixation, growth plate stapling, osteotomy, spinal surgery, arthroplasty) Pharmacological treatment (e.g. oral phosphate and/or active vitamin D, ergocalciferol, cholecalciferol, burosumab, pain medication, cinacalcet, opioids) Attempted surgical resection of tumor % of patients in which resection is successful % of patients in which tumor recurs Time from onset of symptoms to diagnosis Time from symptom onset to successful cure Time from surgery to successful cure Misdiagnosed conditions Laboratory investigations (e.g. frequency and results of biochemical assessments including serum phosphate, calcium, PTH, ALP, bone ALP, FGF23, urinary calcium excretion, 1,25-dihydroxyvitamin D, 25-hydroxyvitamin D, TmP/GFR) Complications typically associated with oral phosphate and active vitamin D treatment (e.g. nephrocalcinosis, kidney stones), hyperparathyroidism, parathyroidectomy, Complications related to the tumor (e.g. functional impairments, complications of tumor removal) Mobility aids/home modifications Absenteeism/presenteeism/physical therapy/caregiving/mental health care Health-related quality of life Costs (e.g. caregiver costs, patient costs, societal costs)	No relevant outcomes reported
Study design	Any studies reporting clinical, humanistic, or economic burden of disease data (e.g. case reports, RWE, observational studies)	Reviews/editorials/commentaries SLR/NMA^a^ In vitro/animal studies/pre-clinical studies
Date limits	Full-text publications 1995 to present (conference abstracts 2022 to present)	Full-text publications pre-1995 (conference abstracts pre-2022)
Countries	No restriction	–
Languages	English language publications	Non-English language publications

ALP, alkaline phosphatase; FGF23, fibroblast growth factor 23; NMA, network meta-analysis; PTH, parathyroid hormone; RWE, real-world evidence; SLR, systematic literature review; TIO, tumor-induced osteomalacia; TmP/GFR, ratio of tubular maximum reabsorption rate of phosphate to glomerular filtration rate

^a^Relevant SLRs/NMAs were included at title/abstract screening stage so their bibliographic reference lists could be hand-searched for relevant studies

### Data Sources

#### Electronic Databases

Searches were conducted on March 20th, 2025 in the following electronic databases: Embase, MEDLINE Daily, In-Process & Other Non-indexed citations, and e-pub ahead-of-print; Cochrane library – Cochrane Central Register of Controlled Trials and Cochrane Database of Systematic Reviews.

#### Hand-Searching

Bibliographic reference lists of included studies and of relevant SLRs/meta-analyses identified during the project were hand searched, as a supplementary measure to ensure that all relevant studies were included in the literature review.

### Search Strategies

The electronic database search strings for the literature review are available in Supplementary Material 1 [18]. The search strings were designed in Embase and translated for the other databases. The searches combined free text and controlled vocabulary terms (Medical Subject Headings [MeSH] in MEDLINE and Emtree terms in Embase).

### Study Selection

Titles/abstracts were screened by a single reviewer against pre-defined selection criteria. Full-text papers were screened by a single reviewer against the selection criteria to ensure the methodology and results were relevant. A record was kept of all publications excluded at this stage, along with a clear justification for their exclusion (based on the pre-defined eligibility criteria).

The earliest full-text manuscript reporting the primary outcome for any study was designated the primary publication. Other citations for the same study were termed ‘linked’ citations. Linked citations that offered no unique information that were superseded by either earlier or later publications were excluded during screening. Linked citations offering unique information were included.

Results from the database searches were downloaded into Covidence®, where duplicates were removed. Covidence® was used to manage citation screening during the title/abstract and full-text screening phases.

### Data Extraction

- Data from included publications were extracted by one reviewer into standardized data extraction tables (DET) in Microsoft^®^ Excel. The information was quality checked by a second independent reviewer.
- The following assumptions/interpretations were made:
- Tumor size was determined by the largest recorded dimension (cm).
- Individuals with ‘impaired physical function’ included those who were bedridden, unable to walk, required ambulatory assistance, had abnormal gait for age, were unable to perform activities, or had falls.
- ‘Hip/pelvis’ in tumor location, and ‘hip’ in fracture location, included the femoral neck, trochanteric/intertrochanteric area, and proximal femur.
- “Biopsy of lesion/tumor” was recorded if the biopsy was done to identify the tumor causing TIO. “Bone biopsy” referred to any bone biopsy (including, but not limited to, trans-iliac histomorphometry), except for that of a lesion/tumor.
- For this review, successful tumor resection was interpreted as biochemical cure and/or symptom resolution. While the definition of successful surgery may more strictly imply correction of biochemical parameters in clinical practice, not all included studies explicitly reported biochemistry results post-surgery. Therefore, a broader definition was used to avoid under-reporting of the success rate.
- Positron emission tomography (PET) scans were categorized as “PET” or “PET/CT” when no other information was provided regarding DOTATATE or fluorodeoxyglucose (FDG) agents. Single-photon emission computed tomography (SPECT) scans included sestamibi scans (e.g. technetium ^99^Tc sestamibi and ^111^indium-pentetreotide scans) and SPECT/CT scans. Dual-energy x-ray absorptiometry (DXA) scans were assessed regarding bone densitometry.
- In cases where it was unclear whether the patient had one or multiple scans or fractures listed, the most conservative value was selected.
- Where time measurements were not precisely specified, the following interpretation was applied:
  - Expressions such as “≤10 days” were assumed to represent 10 days.
  - Expressions such as “<10 days” were assumed to represent 9 days.

### Statistical Analysis

As this was a descriptive study, continuous variables were used and summarized as mean, median, SD and range, using Microsoft® Excel. Categorical variables were summarized as number and percentage.

## Results

### Characteristics of Included Studies

**Fig. 1:**
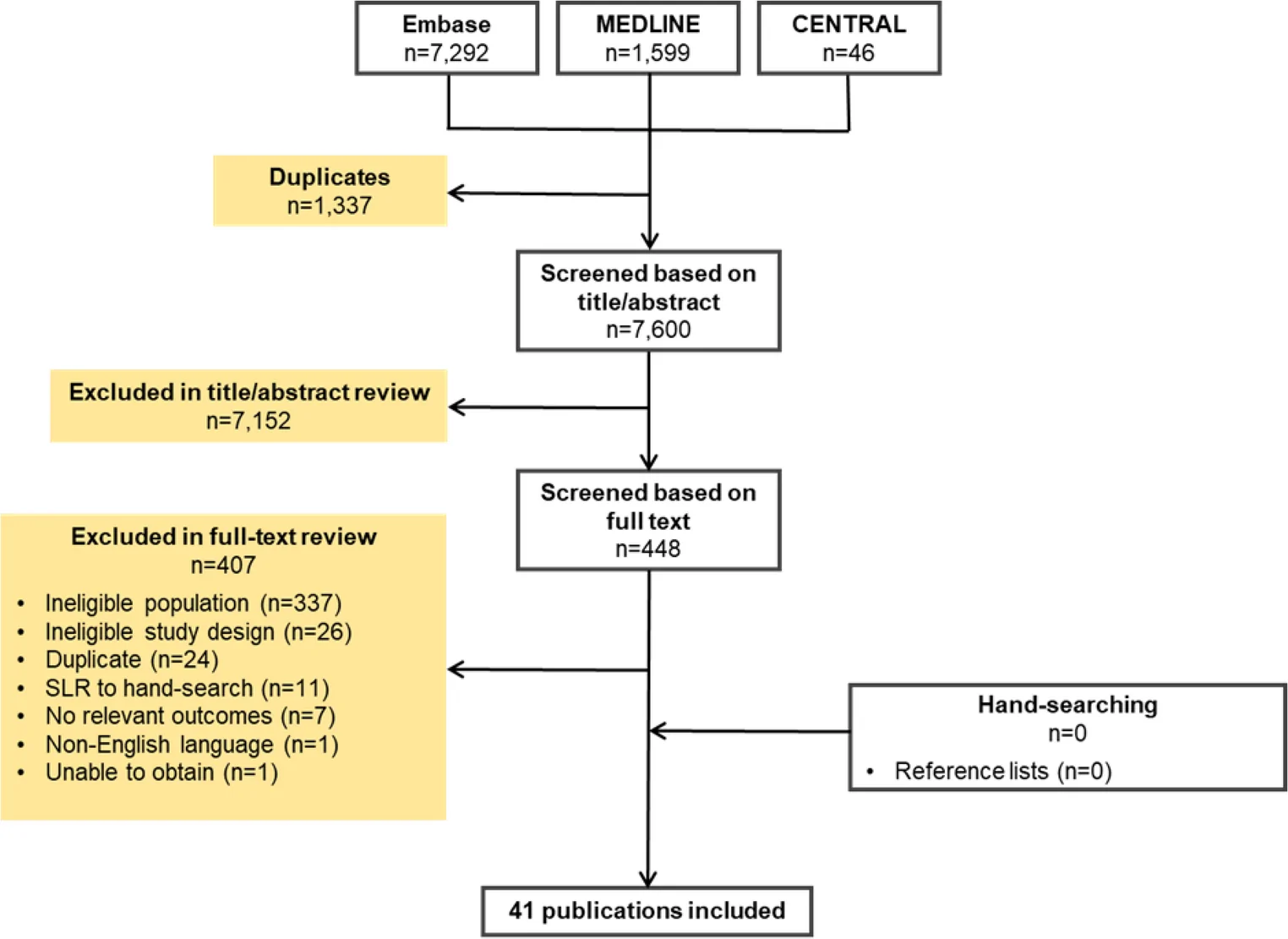
PRISMA diagram for the selection of eligible publications identified through Embase, MEDLINE and CENTRAL. Abbreviations: PRISMA, Preferred Reporting Items for Systematic Reviews and Meta-Analyses; SLR,systematic literature review

The PRISMA (Preferred Reporting Items for Systematic Reviews and Meta-Analysis) diagram for the selection of eligible publications is shown in Fig. 1. In total, 8937 publications were identified through the electronic database searches. After the removal of 1337 duplicates, 7600 publications were reviewed based on their titles and abstracts. A total of 7152 publications were excluded at the title/abstract review stage, leaving 448 potentially relevant publications which were procured for full-text review. After reviewing the full-text publications, a further 407 were excluded, resulting in a total of 41 publications for final inclusion in the review. The 41 publications reported on 46 patients, with the date range of publications spanning 1999 to 2024 (see Supplementary Material 2).

### Patient Characteristics

Patients had a mean age at presentation of 11.2 years (n = 46; standard deviation [SD]: 4.6 years; median age: 12.0 years; range: 1.5–17 years). The majority of patients (n/N = 28/46; 60.9%) were male, 17/46 (36.9%) were female, and 1/46 (2.2%) had no gender reported. A total of 14 countries were represented, with the majority of studies from the USA (14/46; 30.4%), China (10/46; 21.7%), and India (6/46; 13.0%) (Table 2).

**Table 2:** Patient and study characteristics

Patient and study characteristics	*N* = 46
Age (years)	
Mean (SD)	11.2 (4.6)
Median (range)	12 (1.5–17)
Sex n, (%)	
Male	28 (60.9)
Female	17 (36.9)
Missing	1 (2.2)
Country n, (%)	
USA	14 (30.4)
China	10 (21.7)
India	6 (13.0)
Europe^a^	6 (13.0)
Japan	4 (8.7)
Asian region^b^	4 (8.7)
Canada	1 (2.2)
Israel	1 (2.2)

NR, not reported; SD, standard deviation; UK, United Kingdom; USA, United States of America

^a^Included manuscripts from Spain, Austria, Belgium, Switzerland, and the UK

^b^Included manuscripts from Korea, Thailand, and Vietnam

### Disease Manifestations

The most commonly reported symptoms at presentation were pain (30/46; 65.2%), followed by weakness (22/46; 47.8%), and impaired physical function (20/46; 43.5%). Fractures were reported in 16/46 patients (34.8%), where the mean number of fractures per patient was 2.7 (n = 12; SD: 2.3; median: 2; range: 1–9). The location of fracture(s) was reported in 9/16 patients (56.3%). The most common fracture location was the upper leg/femur, recorded in 5/9 patients (55.5%). Rickets was reported in 21/46 patients (45.7%) (Table 3), all of whom were less than 16 years of age at presentation.

The use of a mobility aid (wheelchair, scooter, crutched, cane, and/or walker) was reported in 7/46 patients (15.2%). Impaired activities of daily living due to TIO (such as missing school, being bedridden, or having multiple admissions to hospital) were reported for 6/46 patients (13.0%).

**Table 3:** Disease manifestations

Description	*N* = 46
Manifestations at presentation, n (%)	
Pain	30 (65.2)
Weakness	22 (47.8)
Impaired physical function	20 (43.5)
History of fracture/pseudofracture	8 (17.4)
Lower limb deformity	8 (17.4)
Impaired growth/short stature	5 (10.9)
Fatigue	3 (6.5)
Height loss	2 (4.3)
Patients with rickets, n (%)	21 (45.7)
Patients with a reported fracture, n (%)	16 (34.8)
Number of fractures per patient^a^	*N* = 12
Mean (SD)	2.7 (2.3)
Median (range)	2 (1–9)
Site of fracture for patients with recorded fracture location, n (%)	*N* = 9
Lower limb	
Upper leg/femur	5 (55.5)
Lower leg/tibia/fibula	2 (22.2)
Foot/ankle	1 (11.1)
Trunk	
Rib	2 (22.2)
Spine	1 (11.1)
Upper limb	
Shoulder/arm	2 (22.2)
Hand/wrist	1 (11.1)
Cranial	1 (11.1)

SD, standard deviation

^a^While fractures were reported in 16 patients, only 12 patients had the number of fractures reported

### Diagnosis and Investigations

Time from onset of symptoms to diagnosis was reported in 7/46 patients (15.2%). In those patients, the mean time from onset of symptoms to diagnosis was 4.3 years (SD: 2.6; range: 1–9 years) (Table 4). In the majority of patients, tumors were first detected by imaging +/− clinical examination, biopsy, or lab investigations (35/46; 76.1%). TIO symptoms that were attributed to other conditions were reported for 7/46 patients (15.2%): patients were misdiagnosed with XLH, transient synovitis, bronchitis (tumor was located in the extrapleural space of the right lung), bilateral patellofemoral syndrome and pes planus, myositis ossificans and vitamin D-dependent rickets, Osgood-Schlatter disease, and juvenile idiopathic arthritis (*n* = 1 for each).

The most common laboratory investigations with reported results for initial measurements were: serum phosphate (*n* = 37; mean: 1.8 mg/dL; SD: 0.7), serum total alkaline phosphatase (ALP; *n* = 23; mean: 1,027.9 U/L; SD: 958.7), serum parathyroid hormone (PTH; *n* = 23; mean: 70.8 pg/mL; SD: 61.5); serum calcium (*n* = 20; mean: 9.0 mg/dL; SD: 1.3), and serum 1,25(OH)_2_D (*n* = 20; mean: 22.9 pg/mL; SD: 16.5) (Table 4). The following tests and results were reported in ≤ 5 patients: serum beta-C-terminal telopeptide (β-CTX) (*n* = 4; mean: 1.64 ng/mL, SD: 0.88); serum bone-specific ALP (reported in U/L: *n* = 2, mean: 598.5, SD: 385.37; reported in µg/L: *n* = 1, mean: 36.5), and random urinary calcium/creatinine ratio measurement (*n* = 2; mean: 0.23 mg/mg, SD: 0. 11).

In all patients (37/37) with a reported serum phosphate measurement at presentation, the initial concentration was below the normal range for their age. There were an additional 6/46 patients with no levels reported, but with a diagnosis of hypophosphatemia. Therefore, overall, hypophosphatemia was reported in 43/46 (93.5%) of patients at presentation. Only one patient out of 46 (2.2%), aged 3 years, was reported as having normal phosphate concentration at presentation (with no value reported), although this changed 6 months later where their serum phosphate concentration was 2.6‍ mg/dL (reference range for age: 3.7–5.3 mg/dL). The remaining two patients had no serum phosphate measurements and/or hypophosphatemia reported. Serum ALP, serum intact FGF23 and TRP were all reported as abnormal in 100% of patients with corresponding test results.

Selective venous sampling was performed in 6/46 patients (13.0%), which successfully identified or confirmed the location of the tumor in 5/6 patients (83.3%). Biopsy of lesion/tumor was reported in 19/46 patients (41.3%), while an iliac crest/transiliac bone biopsy was reported in 3/46 patients (6.5%). In 2/3 patients, the bone biopsy showed severe osteomalacia, while the third patient was reported to have had a “normal bone biopsy” at presentation. Of the two patients with severe osteomalacia, one patient, aged 11, had a mean osteoid thickness of 714% of the upper limit of normal (ULN) for their age, and the other, aged 4, 506% of the age-related ULN (normal range not reported in the second publication but taken from Glorieux et al., 2000 [19]). Mineralization lag time was not reported, with one study reporting that the lag time was not calculable due to the severity of osteomalacia.

Genetic testing was reported in 11/46 patients (23.9%), investigating the following genes: phosphate regulating endopeptidase X-linked (*PHEX*; 6/11; 54.5%), *FGF23* (4/11; 36.4%), dentin matrix acidic phosphoprotein 1 (*DMP1;* 3/41, 27.3%), ectonucleotide pyrophosphatase/phosphodiesterase 1 (*ENPP1;* 2/11, 18.2%), unspecified genes linked to XLH (2/11, 18.2%), SH3 domain binding protein 2 (*SH3BP2*; 1/11, 9.1%) and unspecified genes linked to cherubism (1/11, 9.1%). Whole exome sequencing was performed in 2/11 patients (18.2%). No pathogenic variants were detected; however, two variants of unknown significance were identified in one patient: a heterozygous transversion in exon 6 of the *DMP1* gene (c.475 C > A, p.Gln159Lys), and another heterozygous transversion in the promoter region of *ENPP1* (c.–24 G > C). Overall, no patients had a reported family history of hypophosphatemia or metabolic bone disease, although a lack of family history was expressly stated in only 9/46 patients (19.6%).

Imaging procedures were reported in 41/46 patients (89.1%). The mean number of imaging procedures per patient was 5.4 (SD: 2.7; range: 2–12). The most commonly reported imaging tests were X-rays (28/41; 68.3%), magnetic resonance imaging (MRI; 24/41; 58.5%), and computed tomography (CT; 24/41; 58.5%) (Table 4).

**Table 4:** Diagnosis and disease investigations

Description	*N* = 46
Time from onset of symptoms to diagnosis, years	
N	7
Mean (SD)	4.3 (2.6)
Median (range)	4 (1–9)
Patients misdiagnosed, n (%)	7 (15.2)

Laboratory investigations	n^a^	Mean (SD)	n (%) below/above normal range
Serum phosphate (mg/dL)^b^	37	1.8 (0.7)	37 (100) below
Serum calcium (mg/dL)	20	9.0 (1.3)	3 (15.0) below
Serum creatinine (mg/dL)	6	0.55 (0.1)	1 (16.7) below
Serum ALP (U/L)	23	1,027.9 (958.7)	23 (100) above
Serum PTH (pg/mL)	23	70.8 (61.5)	8 (34.8) above
Serum 1,25(OH)_2_D (pg/mL)	20	22.9 (16.5)	12 (57.1) below
Serum 25-hydroxyvitamin D (ng/mL)	19	22.2 (10.5)	5 (26.3) below
Serum intact FGF23 (pg/mL)	13	374.5 (355.9)	13 (100) above
Serum C-term FGF23 (RU/mL)	13	579.1 (551.0)	11 (84.6) above
TRP (%)	10	69.3 (24.7)	10 (100) below
TmP/GFR (mmol/L)	17	0.3 (0.2)	15 (93.8) below
Selective venous sampling for FGF23, n (%)		6 (13.0)	
Imaging procedures		*N* = 41	
Mean number of procedures (SD)		5.4 (2.7)	
Median number of procedures (range)		4.0 (2–12)	
Type of imaging procedures, n (%)		*N* = 41	
X-ray		28 (68.3)	
MRI		24 (58.5)	
CT		24 (58.5)	
Octreotide scan		14 (34.1)	
Technetium bone scan		11 (26.8)	
DXA scan		10 (24.4)	
Ultrasound		3 (7.3)	
SPECT		2 (4.9)	
PET scans, n (%)			
PET/CT		3 (7.3)	
FDG PET/CT		6 (14.6)	
DOTATATE/ DOTANOC PET/CT		10 (24.4)	
Biopsy, n (%)			
Bone biopsy		3 (6.5)	
Biopsy of lesion/tumor		19 (41.3)	

1,25(OH)_2_D, 1,25-dihydroxyvitamin D; ALP, alkaline phosphatase; CT, computed tomography; DXA, dual-energy x-ray absorptiometry; FDG, fluorodeoxyglucose; FGF23, fibroblast growth factor 23; MRI, magnetic resonance imaging; PET, positron emission tomography; PTH, parathyroid hormone; SD, standard deviation; SPECT, single-photon emission computed tomography; TmP/GFR, ratio of tubular maximum reabsorption rate of phosphate to glomerular filtration rate

^a^n represents patients with a recorded value for the test result; there may have been more patients with a reported test but no recorded result

^b^ Age- and sex- specific pediatric reference serum phosphate values are available in Supplementary Material 3 [18]

### Tumor Characteristics

**Fig. 2:**
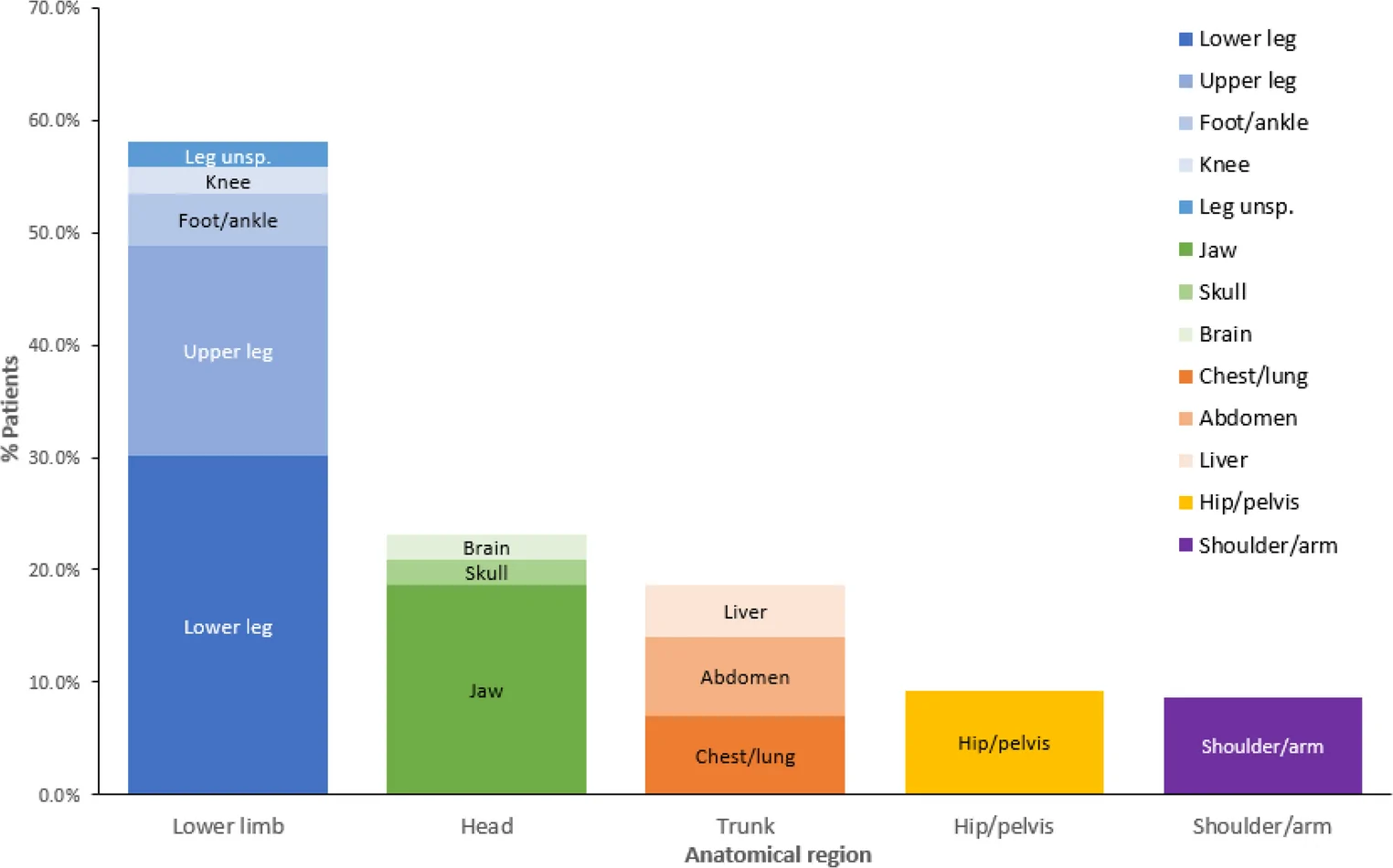
Visual representation of tumor location by anatomical region and individual site. In the case report studies, tumor locations were reported by site, which are further grouped by anatomical region here. Please note: some patients may have tumors in multiple locations . Abbreviations: unsp., unspecified

The location of the tumor was reported in most individuals (43/46; 93.5%). The most commonly reported tumor locations were the lower leg (13/43; 30.2%), followed by the head (10/43; 23.3%), and upper leg (8/43; 18.6%) (Fig. 2; Table 5). Tumor size was reported in 25/46 patients (54.3%). The mean reported tumor size (largest dimension) was 5.5 cm (SD: 4.2; range: 1.5–15.5).

**Table 5:** Tumor characteristics

Description	
Patients with known tumor location, n (%)	*N* = 43
Lower limb	
Lower leg	13 (30.2)
Upper leg	8 (18.6)
Foot/ankle	2 (4.7)
Knee	1 (2.3)
Leg unspecified	1 (2.3)
Hip/pelvis	4 (9.3)
Head	
Jaw	8 (18.6)
Skull	1 (2.3)
Brain	1 (2.3)
Trunk	
Abdomen	3 (7.0)
Chest/lung	3 (7.0)
Shoulder/arm	4 (9.3)
Liver	2 (4.7)
Metastatic tumor, n (%)	*N* = 46
Yes	3 (6.5)
Tumor size, cm^a^	*N* = 25
Mean (SD)	5.5 (4.2)
Median (range)	4 (1.5–15.5)
% of patients within each tumor size stratum, n (%)^a^	*N* = 25
< 1.5 cm	1 (4.0)
1.5–2.5 cm	7 (28.0)
2.6–4.0 cm	7 (28.0)
> 4.0 cm	10 (40.0)

SD, standard deviation

Tumor histology was available for 40/46 patients (87.0%). In those patients, the most commonly reported type of tumor was phosphaturic mesenchymal tumor (PMT) (26/40; 65.0%). Other tumor types included giant cell granuloma (2/40; 5.0%), osteosarcoma (2/40; 5.0%), and ossifying fibroma (2/40; 5.0%). In addition, hepatoblastoma, aneurysmal bone cyst, fibro-osseous lesion, malignant fibrosarcoma, undifferentiated embryonal sarcoma of the liver, hemangiopericytoma, hepatic hemangioendothelioma and renal clear cell carcinoma were also reported (each 1/40; 2.5%), potentially indicating secondary TIO in some cases. Patients with PMTs were older (n = 26, mean age: 12.4 years; SD: 4.1) than those with other types of tumor (n = 14, mean age: 8.8 years; SD: 5.22). Initial phosphate measurement was higher in patients with PMT (n = 19; mean: 1.93, SD: 0.67) than in other tumors (n = 12; mean: 1.75, SD: 0.82).

Presence of a metastatic tumor was reported in 3/46 patients (6.5%). One of the metastatic tumors was a high-grade malignant fibrosarcoma of the distal femur, with lung nodules at presentation that were suggestive of lung metastases, later confirmed as such. Another metastatic tumor originally presented as multiple PMTs in the leg and lungs, with no malignant features; three years after resection of the benign PMTs, a pleomorphic sarcoma was found in the left leg, which was considered to be a malignant transformation of the PMT. The third metastatic tumor also presented as a benign PMT (of mixed connective tissue variant [MCT]) of the trapezius; 4 months following resection, new lesions emerged in both legs, arm and lungs. All biopsied specimens showed a histology of PMT-MCT, identical to that of the primary tumor. Three years later, the patient developed a pleomorphic sarcoma in her right thigh, with multiple metastases to the inguinal lymph nodes, pancreas, right ovary and brain.

### Treatment

#### Tumor Removal

Attempted surgical resection was reported in the majority of patients (41/46; 89.1%). There were no reported cases of radiofrequency ablation (RFA) (Table 6). In the 5/46 patients with no resection attempt, tumor location was not documented for two patients, tumor was unresectable in one patient, and there was no information regarding surgery for the remaining two patients. In patients with a resection attempt, the number of resections was reported for 35/41 (85.4%) patients. Mean number of resection attempts per patient was 1.2 (SD: 0.5); 6/35 patients (17.1%) underwent more than one surgical resection attempt. Successful resection was reported in 28/46 patients (60.9%), while tumor recurrence was reported in 2/46 patients (4.3%), both patients previously had multiple benign PMTs resected. In patients with successful resection, normalized biochemical parameters were reported in 18/28 patients (64.3%); for 6/28 patients (21.4%), successful cure was reported as an improvement/resolution of clinical symptoms and/or radiological findings. For 4/28 patients (14.3%), the definition of successful cure was not reported. Mean follow-up after surgery was 1.2 years (SD: 1.0).

**Table 6:** Treatment of disease

Description	*N* = 46
Removal of lesion	
Surgical resection attempted, n (%)	41 (89.1)
Overall successful resection of tumor, n (%)	28 (60.9)
Incomplete resection, n (%)	7 (15.2)
Patients with known no. of resection attempts	*N* = 35
Mean (SD)	1.2 (0.5)
Median (range)	1 (1–3)
No. of patients with > 1 resection attempt, n (%)	6 (17.1)
Tumor recurrence, n (%)	2 (4.3)
Supplementation and pharmacologic treatment, n (%)	
Oral phosphate	23 (50.0)
Active vitamin D	21 (45.7)
Over-the-counter vitamin D	9 (19.6)
Calcium	6 (13.0)
Cinacalcet	2 (4.3)
Pain medication	1 (2.2)
Orthopedic procedures, n (%)	
Surgical fracture fixation	1 (2.2)
Knee arthroplasty	1 (2.2)
Other^a^	2 (4.3)
Complications associated with oral phosphate and active vitamin D, n (%)^b^	
Hyperparathyroidism	3 (6.5)

^a^Includes partial maxillectomy with free fibula flap reconstruction (*n* = 1) and left distal femoral medial hemiepiphysiodesis (*n* = 1)

^b^Complications associated with, not necessarily caused by, oral phosphate and active vitamin D treatment

SD, standard deviation

### Pharmacologic Treatment and Surgical Procedures

The most commonly reported pharmacologic treatments were oral phosphate supplementation (23/46; 50.0%) and active vitamin D (21/46; 45.7%) (Table 6). No patients were treated with burosumab; over two thirds of the included studies were published before the earliest commercial availability of burosumab [16]. Overall, 3/46 patients (6.5%) developed secondary hyperparathyroidism, which was described as potentially due to oral phosphate supplementation in two out of those three patients. No cases of nephrocalcinosis were reported.

With regard to orthopedic procedures, surgical fixation of fracture, knee arthroplasty, partial maxillectomy with free fibula flap reconstruction, and left distal femoral medial hemiepiphysiodesis were reported in 1/46 patient each (2.2%).

## Discussion

To our knowledge, this review is the first to assess the burden of illness in pediatric patients with TIO. Our review found that TIO poses a substantial burden to the health and well-being of children. As in adults, pain was reported by the majority of children (65.2%), while weakness was reported in just under half (47.8%) and impaired physical function in 43.5% of patients. Rickets, a finding specific to pediatric-onset disease, was observed in 45.7% of the patients in this review. The most common fracture location was the upper leg/femur, a location which is typically associated with considerable morbidity, and is relatively uncommon in children without TIO [20, 21]. Mean time from onset of symptoms to diagnosis was 4.3 years (*n* = 7); in pediatric patients, a 4-year delay in diagnosis can be more than 50–75% of the lifespan and may lead to irreversible manifestations. Misdiagnoses were reported in 15.2% of patients (including XLH, but also more common pediatric musculoskeletal disorders such as transient synovitis, patellofemoral syndrome, pes planus and Osgood-Schlatter disease, among others). A previous study of TIO in adults showed that longer time to diagnosis was associated with poorer tumor resection outcomes and a higher probability of tumor recurrence [11]. While the small sample size in this study precluded any association analyses from being performed, it is possible that delays in diagnosis may also lead to poorer outcomes in pediatric age groups. Also, the prolonged interval between symptom onset and diagnosis means that children may undergo repeated radiographic and CT imaging, raising concerns this could inadvertently increase cumulative radiation exposure in a vulnerable pediatric population: in this review, the mean number of imaging procedures per patient was 5.4. Additionally, 41.3% of patients had a biopsy of the suspected TIO lesion, which carries a risk of tumor dissemination and could worsen disease outcomes. Thus, a multidisciplinary stepwise approach is required for timely diagnosis in all cases of pediatric hypophosphatemia to determine the mechanism of phosphate deficiency and explore the possibility of causal treatment as early as possible [1, 22].

As expected, the majority of children (93.5%) had low serum phosphate levels at presentation; in those with reported initial test results, 100% had ALP levels above the normal range and 100% had TRP levels below the normal range. Over a third (34.8%) of patients had elevated PTH, an interesting finding, which is consistent with the reported frequencies of secondary hyperparathyroidism in patients with XLH, suggesting this is an FGF23 mediated phenomenon. Intact FGF23 levels were elevated in 100% of patients, and C-terminal FGF23 in 84.6%: this is in keeping with previous studies that suggest elevations of intact FGF23 are more sensitive to detecting TIO (or other FGF23-mediated hypophosphatemia) than C-terminal FGF23 [14, 23]. The majority of patients (73.7%) had normal 25-hydroxyvitamin D levels, while 57.1% had 1,25(OH)_2_D levels below the normal range: interestingly, the only patient who was misdiagnosed with vitamin-D deficient rickets had neither 25-hydroxyvitamin D or 1,25(OH)_2_D levels reported.

Almost two-thirds of tumors in this review were PMTs; this is particularly interesting, as PMTs are typically seen in middle-aged adults, with a median age at the time of diagnosis of 44–48 years [24]. The frequency of metastatic tumors in this review (6.5%) was slightly higher than that observed (4.4%) in a previous literature review of TIO reporting on a predominantly adult (95%) population, while tumors were on average 2.4 cm larger (5.5 cm vs. 3.1 cm) [11]. Where the causative tumor(s) had been localized, surgical resection was attempted in the majority of patients (89.1%), similar to previous reviews of predominantly adult populations where surgical resection was attempted in 81.0–98.7% of patients [11, 25–27]. The rate of successful resection (60.9%) in this review of pediatric cases is lower than the rates reported in adults (67.0–94.0%) [11, 26, 27], and although the definition of successful surgery varies greatly between publications, this means that surgery was unsuccessful in almost 40% of patients. In this review, the mean duration of follow-up after surgery was only 1.2 years (SD: 1.0), making it difficult to estimate long-term cure rates in a pediatric population. Patients with pediatric-onset TIO who did not receive treatment until after reaching skeletal maturity may continue to experience a heavy symptomatic burden indefinitely, due to skeletal deformities and short stature.

Approximately half the patients were reported to have received oral phosphate supplementation (50.0%) and/or active vitamin D (45.7%). Secondary hyperparathyroidism was reported in 6.5% of patients, and this was potentially linked to oral phosphate supplementation in two thirds of this group. Secondary hyperparathyroidism appears to be more prevalent in adults with TIO, with a study finding 17% developed the complication despite not being on phosphate supplementation, possibly due to the suppressive effects of chronic elevations of FGF23 on endogenous calcitriol production [28]. No patients were treated with burosumab in the included studies as most reports were published before burosumab was commercially available. However, burosumab use may increase in the future, which could affect musculoskeletal outcomes in patients with unresectable tumors; clinical studies have shown improvements in pain/stiffness/physical function and healing of fractures in patients treated with burosumab. Regardless, neither burosumab nor phosphate/active vitamin D treat the underlying tumor or prevent tumor progression. Rather, these forms of medical intervention serve to treat osteomalacia (and rickets, in children), particularly when the causative tumor cannot be resected, or to promote bone healing prior to surgery.

The possibility of previously undetected genetic abnormalities should also be considered, as certain genetic variants may result in the clinical features observed in TIO. In this review, genetic testing was only reported in 11 patients (23.9%), two of whom underwent whole exome sequencing; in the remaining nine patients, only genes related to XLH and/or cherubism were sequenced. When no tumor can be localized, and no genetic etiology is identified, consideration should be given to autoimmune disease, in view of recent reports of autoantibodies to PHEX resulting in hypophosphatemic osteomalacia [29].

### Limitations

This study has several limitations, including the scarcity of data when examining a rare disease. Furthermore, there is likely under-reporting in the literature and studies vary considerably with respect to the presented information. For example, a key outcome of our study, mean time from onset of symptoms to diagnosis, was reported in only 15.2% of patients. With regards to laboratory investigations, values were described in some reports as normal or abnormal (or not commented upon), but with no definitive value presented. Despite the majority (93.5%) of patients having low serum phosphate concentrations at presentation, phosphate supplementation was only reported in 50% of patients; similarly, use of analgesia was only reported in one patient (2.2%), despite pain being the most common symptom at presentation (65.2% of patients). Thus, details of manifestations and treatments may not be fully represented, as our review is reliant on secondary information subject to selectivity of the primary reports. It seems likely that under-reporting of symptoms may lead to an underestimation of the true burden of illness in pediatric TIO; patient-reported outcomes were reported in only one study, and only 6/46 studies (13.0%) reported the impact TIO had on activities of daily living, such as school attendance.

As inferred above, the included publications may be subject to bias, thus limiting generalizability. Case reports may be more likely to focus on noteworthy cases, and cases without confirmed tumor localization may be less likely to be published, therefore overestimating the true number of patients in which the causative tumor was successfully localized. In our review, 91.3% of patients had the causative tumor localized, compared with 60–65% reported in adult cases of TIO [13, 14].

The included publications were from several countries, which may have contributed to heterogeneity of data across different patient populations and variance in reported outcomes. Additionally, some laboratory investigations are age-dependent (e.g. serum phosphate levels are higher in children than in adults), and therefore normal levels reported between publications differed, making direct comparisons challenging [3]. Finally, although this review only included patients with a diagnosis of TIO, it was not possible to definitively confirm the diagnosis by biochemical assessment in all cases, due to non-standardized diagnosis reporting across studies.

## Conclusion

Pediatric-onset TIO is associated with a substantial symptomatic and healthcare burden. Common manifestations such as rickets may be attributed to other more common causes, leading to delays in diagnosis and excessive investigations, including radiation-conferring tests. Further research and extended periods of observation are needed to provide a more complete and long-term picture of the clinical burden in childhood TIO.

## Supplementary Information

Below is the link to the electronic supplementary material.

Supplementary Material 1

## Data Availability

Some or all datasets generated during and/or analyzed during the current study are not publicly available but are available from the corresponding author on reasonable request.
