# Supplementary material for: Burden of Disease in Pediatric Tumor-Induced Osteomalacia: A Literature Review
Source: Calcif Tissue Int. 2026 Aug 10;117(1):126. doi: 10.1007/s00223-026-01580-0 (PMC13457281; doi:10.1007/s00223-026-01580-0)
Supplement: Supplementary file 1 [file 223_2026_1580_MOESM1_ESM.docx]

Burden of disease in pediatric tumor-induced osteomalacia: a literature review

Supplementary material

1. Literature review methods
   1.1. Search string
        1.1.1. Medline search string

Databases: **Ovid MEDLINE(R) ALL** 1946 to March 19, 2025

Date searched: 20/03/2025

Hits: 1,599

Table 1: Medline search string

| # | Searches | Results |
| --- | --- | --- |
| 1 | exp Osteomalacia/ | 5125 |
| 2 | exp Hypophosphatemia/ | 4426 |
| 3 | (hypophospat?emi* or rickets).tw. | 8002 |
| 4 | (((tumo?r or cancer* or onco*) adj3 osteomalacia*) or (TIO and osteo*)).tw. | 1172 |
| 5 | (osteomalacia* or osteo-malacia*).af. | 7779 |
| 6 | exp Neoplasms/ | 4087557 |
| 7 | (tumo?r or neoplas* or onco* or cancer*).tw. | 3755991 |
| 8 | (1 or 2 or 3 or 5) and (6 or 7) | 2281 |
| 9 | 4 or 8 | 2424 |
| 10 | animals/ | 7625768 |
| 11 | exp animals, laboratory/ | 991912 |
| 12 | exp animal experimentation/ | 10673 |
| 13 | exp models, animal/ | 677136 |
| 14 | exp rodentia/ | 3695756 |
| 15 | (rat or rats or mouse or mice).ti. | 1498273 |
| 16 | or/10-15 | 7755724 |
| 17 | humans/ and 16 | 2349215 |
| 18 | 16 not 17 | 5406509 |
| 19 | 9 not 18 | 2337 |
| 20 | exp editorial/ or exp news/ | 953144 |
| 21 | 19 not 20 | 2319 |
| 22 | limit 21 to english language | 1969 |
| 23 | limit 22 to yr="1995 -Current" | 1599 |

1.1.2. Embase search string

Databases: **Embase** 1974 to 2025 March 19

Date searched: 20/03/2025

Hits: 7,292

Table 2: Embase search string

| **#** | **Searches** | **Results** |
| --- | --- | --- |
| 1 | exp osteomalacia/ | 11724 |
| 2 | exp oncogenic osteomalacia/ | 1112 |
| 3 | exp hypophosphatemia/ | 15176 |
| 4 | (((tumor or cancer* or onco*) adj3 osteomalacia*) or (TIO and osteo*)).tw. | 1473 |
| 5 | (osteomalacia* or osteo-malacia*).af. | 10849 |
| 6 | (hypophospat?emi* or rickets).tw. | 9058 |
| 7 | exp neoplasm/ | 6230812 |
| 8 | (tumor or neoplas* or onco* or cancer*).tw. | 4985273 |
| 9 | (1 or 2 or 3 or 5 or 6) and (7 or 8) | 9963 |
| 10 | 4 or 9 | 10059 |
| 11 | animal/ | 1693456 |
| 12 | nonhuman/ | 8068299 |
| 13 | exp animal experiment/ | 3324588 |
| 14 | exp experimental animal/ | 884176 |
| 15 | animal model/ | 1891309 |
| 16 | exp rodent/ | 4277210 |
| 17 | (rat or rats or mouse or mice).ti. | 1675695 |
| 18 | or/11-17 | 10636981 |
| 19 | human/ and 18 | 3086666 |
| 20 | 18 not 19 | 7550315 |
| 21 | 10 not 20 | 9881 |
| 22 | exp editorial/ | 802528 |
| 23 | 21 not 22 | 9798 |
| 24 | limit 23 to conference abstract status | 1949 |
| 25 | limit 24 to yr="2022 -Current" | 359 |
| 26 | 24 not 25 | 1590 |
| 27 | 23 not 26 | 8208 |
| 28 | limit 27 to english language | 7703 |
| 29 | limit 28 to yr="1995 -Current" | 7292 |

1.1.3. Cochrane search string

Databases: **EBM Reviews - Cochrane Central Register of Controlled Trials** February 2025**, EBM Reviews - Cochrane Database of Systematic Reviews** 2005 to March 19, 2025

Date searched: 20/03/2025

Hits: 46

Table 3: Cochrane search string

| **#** | **Searches** | **Results** |
| --- | --- | --- |
| 1 | exp Osteomalacia/ | 49 |
| 2 | exp Hypophosphatemia/ | 104 |
| 3 | (hypophospat?emi* or rickets).tw. | 325 |
| 4 | (((tumo?r or cancer* or onco*) adj3 osteomalacia*) or (TIO and osteo*)).tw. | 8 |
| 5 | (osteomalacia* or osteo-malacia*).af. | 225 |
| 6 | exp Neoplasms/ | 126735 |
| 7 | (tumo?r or neoplas* or onco* or cancer*).tw. | 240216 |
| 8 | (1 or 2 or 3 or 5) and (6 or 7) | 58 |
| 9 | 4 or 8 | 61 |
| 10 | limit 9 to english language [Limit not valid in CDSR; records were retained] | 59 |
| 11 | limit 10 to yr="1995 -Current" | 46 |

2. Publications included in the review

Table 4: Case report studies included in review (n=41)

| # | Author year | Full reference |
| --- | --- | --- |
| 1 | Andreopoulou 2011 | Andreopoulou P, Dumitrescu CE, Kelly MH, Brillante BA, Cutler Peck CM, Wodajo FM, et al. Selective venous catheterization for the localization of phosphaturic mesenchymal tumors. Journal of Bone and Mineral Research. 2011;26(6):1295EP-302. |
| 2 | Bhavani 2016 | Bhavani N, Reena Asirvatham A, Kallur K, Menon AS, Pavithran PV, Nair V, et al. Utility of Gallium-68 DOTANOC PET/CT in the localization of Tumour-induced osteomalacia. Clinical Endocrinology. 2016;84(1):134EP-40. |
| 3 | Biswal 2024 | Biswal B, Aradhana A, Mohanty MD, Choudhury J, Behera BK, Beura S. Tumor-Induced Osteomalacia- Like Syndrome with Rickets and Infantile Hepatic Hemangioendothelioma. Indian Journal of Pediatrics. 2024;91(8):842EP-4. |
| 4 | Bowden 2024 | Bowden J, Nasomyont N. Tumor-induced osteomalacia: A rare association with newly diagnosed hepatoblastoma. JBMR Plus. 2024;8(Supplement 2):ii326. |
| 5 | Burckhardt 2015 | Burckhardt MA, Schifferli A, Krieg AH, Baumhoer D, Szinnai G, Rudin C. Tumor-associated FGF-23-induced hypophosphatemic rickets in children: a case report and review of the literature. Pediatric Nephrology. 2015;30(1):179EP-82. |
| 6 | Carter 2015 | Carter JM, Caron BL, Dogan A, Folpe AL. A novel chromogenic in situ hybridization assay for FGF23 mRNA in phosphaturic mesenchymal tumors. The American journal of surgical pathology. 2015;39(1):75EP-83. |
| 7 | Chatterjee 2021 | Chatterjee D, Bardia A, Pal R, Saikia UN, Bhadada SK, Radotra BD. Clinical, morphological and immunohistochemical analysis of 13 cases of phosphaturic mesenchymal tumor - A holistic diagnostic approach. Annals of Diagnostic Pathology. 2021;54:151783. |
| 8 | Chong 2013 | Chong WH, Andreopoulou P, Chen CC, Reynolds J, Guthrie L, Kelly M, et al. Tumor localization and biochemical response to cure in tumor-induced osteomalacia. Journal of Bone and Mineral Research. 2013;28(6):1386EP-98. |
| 9 | Crossen 2017 | Crossen SS, Zambrano E, Newman B, Bernstein JA, Messner AH, Bachrach LK, et al. Tumor-induced osteomalacia in a 3-year-old with unresectable central giant cell lesions. Journal of Pediatric Hematology/Oncology. 2017;39(1):e21EP-e4. |
| 10 | Dhammi 2010 | Dhammi IK, Jain AK, Singh AP, Mishra P, Jain S. Oncogenic osteomalacia: Problems in diagnosis and long-term management. Indian journal of orthopaedics. 2010;44(4):453-7. |
| 11 | Emodi 2018 | Emodi O, Rachmiel A, Tiosano D, Nagler RM. Maxillary tumour-induced osteomalacia. International Journal of Oral and Maxillofacial Surgery. 2018;47(10):1295EP-8. |
| 12 | Eyskens 1995 | Eyskens B, Proesmans W, Van Damme B, Lateur L, Bouillon R, Hoogmartens M. Tumour-induced rickets: A case report and review of the literature. European Journal of Pediatrics. 1995;154(6):462EP-8. |
| 13 | Farmakis 2015 | Farmakis SG, Siegel MJ. Phosphaturic mesenchymal tumor of the tibia with oncogenic osteomalacia in a teenager. Pediatric Radiology. 2015;45(9):1423EP-6. |
| 14 | Fernandez-Cooke 2015 | Fernandez-Cooke E, Cruz-Rojo J, Gallego C, Romance AI, Mosqueda-Pena R, Almaden Y, et al. Tumor-induced rickets in a child with a central giant cell granuloma: A case report. Pediatrics. 2015;135(6):e1518EP-e23. |
| 15 | Haeusler 2010 | Haeusler G, Freilinger M, Dominkus M, Egerbacher M, Amann G, Kolb A, et al. Tumor-induced hypophosphatemic rickets in an adolescent boy - Clinical presentation, diagnosis, and histological findings in growth plate and muscle tissue. Journal of Clinical Endocrinology and Metabolism. 2010;95(10):4511EP-7. |
| 16 | Hasegawa 1999 | Hasegawa T, Shimoda T, Yokoyama R, Beppu Y, Hirohashi S, Maeda S. Intracortical osteoblastic osteosarcoma with oncogenic rickets. Skeletal Radiology. 1999;28(1):41EP-5. |
| 17 | Hou 2022 | Hou G, Zhang Y, Liu Y, Wang P, Xia W, Xing X, et al. Head-to-Head Comparison of 68Ga-DOTA-TATE and 68Ga-DOTA-JR11 PET/CT in Patients With Tumor-Induced Osteomalacia: A Prospective Study. Frontiers in Oncology. 2022;12:811209. |
| 18 | Imel 2006 | Imel EA, Peacock M, Pitukcheewanont P, Heller HJ, Ward LM, Shulman D, et al. Sensitivity of fibroblast growth factor 23 measurements in tumor-induced osteomalacia. Journal of Clinical Endocrinology and Metabolism. 2006;91(6):2055EP-61. |
| 19 | Jacobs 2022 | Jacobs B, Rajakulasingam R, Mughal MZ. Juvenile Psammomatoid Ossifying Fibroma (JPOF) in the Radius: A rare cause of Tumour Induced Osteomalacia. JBMR Plus. 2022;6(Supplement 1):65EP-6. |
| 20 | Jagtap 2011 | Jagtap VS, Sarathi V, Lila AR, Malhotra G, Sankhe SS, Bandgar T, et al. Tumor-induced osteomalacia: A single center experience. Endocrine Practice. 2011;17(2):177EP-84. |
| 21 | Jiajue 2021 | Jiajue R, Ni X, Jin C, Huo L, Wu H, Liu Y, et al. Early Discrimination Between Tumor-Induced Rickets/Osteomalacia and X-Linked Hypophosphatemia in Chinese Children and Adolescents: A Retrospective Case-Control Study. Journal of Bone and Mineral Research. 2021;36(9):1739EP-48. |
| 22 | Jung 2010 | Jung GH, Kim JD, Cho Y, Chung SH, Lee JH, Sohn KR. A 9-month-old phosphaturic mesenchymal tumor mimicking the intractable rickets. Journal of Pediatric Orthopaedics Part B. 2010;19(1):127EP-32. |
| 23 | Kato 2021 | Kato A, Nakamoto Y, Ishimori T, Hayakawa N, Ueda M, Temma T, et al. Diagnostic performance of 68Ga-DOTATOC PET/CT in tumor-induced osteomalacia. Annals of Nuclear Medicine. 2021;35(3):397EP-405. |
| 24 | Khaliq 2011 | Khaliq W, Cheripalli P, Tangella K. Tumor-induced osteomalacia (TIO): Atypical presentation. Southern Medical Journal. 2011;104(5):348EP-50. |
| 25 | Lee 2017 | Lee JY, Park HS, Han S, Lim JK, Hong N, Park SI, et al. Localization of oncogenic osteomalacia by systemic venous sampling of fibroblast growth factor 23. Yonsei Medical Journal. 2017;58(5):981EP-7. |
| 26 | Lerdrassameethad 2024 | Lerdrassameethad W, Promsod O, Charoenlap C, Ariyachaipanich A, Sahakitrungruang T. Unusual Presentation of Tumor-Induced Osteomalacia with Suppressed FGF-23 in an 8-Year- Old Boy. Hormone Research in Paediatrics. 2024;97(Supplement 3):213. |
| 27 | Liu 2019 | Liu SZ, Zhou X, Song A, Huo Z, Wang YP, Liu Y. Tumor-induced osteomalacia caused by a phosphaturic mesenchymal tumor of the femur. Chinese Medical Journal. 2019;132(19):2380EP-1. |
| 28 | Morrell 2014 | Morrell NT, Beck NL, Clericuzio C, Frias-Kletecka C, Szalay EA. Tumor-induced rickets presenting in an adolescent: A case report and review of the literature. JBJS Case Connector. 2014;4(3):e79. |
| 29 | Olivas-Mazon 2020 | Olivas-Mazon R, Martin-Cazana M, Perez-Mohand P, Garzon-Lorenzo L, Espino-Hernandez M, Baro-Fernandez M, et al. Tumor-induced osteomalacia in an adolescent with an undifferentiated embryonal sarcoma of the liver. Pediatric Blood and Cancer. 2020;67(7):e28386. |
| 30 | Oyama 2020 | Oyama N, Kojima-Ishii K, Toda N, Matsuo T, Tocan V, Ohkubo K, et al. Malignant transformation of phosphaturic mesenchymal tumor: A case report and literature review. Clinical Pediatric Endocrinology. 2020;29(2):69EP-75. |
| 31 | Parameshwar 2024 | Parameshwar VC, Rekhi B, Duggad A, Ramadwar M. Phosphaturic mesenchymal tumor: Clinicopathological features with outcomes in 10 patients with review of literature. Indian Journal of Pathology and Microbiology. 2024;67(2):306EP-11. |
| 32 | Reyes-Mugica 2000 | Reyes-Mugica M, Arnsmeier SL, Backeljauw PF, Persing J, Ellis B, Carpenter TO. Phosphaturic mesenchymal tumor-induced rickets. Pediatric and Developmental Pathology. 2000;3(1):61EP-9. |
| 33 | Sakai 2023 | Sakai T, Okuno Y, Murakami N, Shimoyama Y, Imagama S, Nishida Y. Case report: Novel NIPBL-BEND2 fusion gene identified in osteoblastoma-like phosphaturic mesenchymal tumor of the fibula. Frontiers in Oncology. 2023;12:956472. |
| 34 | Shulman 2004 | Shulman DI, Hahn G, Benator R, Washington K, White KE, Farber J, et al. Tumor-induced rickets: Usefulness of MR gradient echo recall imaging for tumor localization. Journal of Pediatrics. 2004;144(3):381EP-5. |
| 35 | Terek 2001 | Terek RM, Nielsen GP. A 14-year-old boy with abnormal bones and a sacral mass. New England Journal of Medicine. 2001;345(12):903EP-8. |
| 36 | Thi 2023 | Thi HN, Manh CP, Tuan LT, Thi LAL, Thanh NN, Vilaiyuk S. Tumor-induced Osteomalacia in a Boy with Maxillary Ossifying Fibroma. JCRPE Journal of Clinical Research in Pediatric Endocrinology. 2023;15(4):421EP-5. |
| 37 | Wang 2018 | Wang L, Zhang S, Jing H, Chen L, Wang Z, Li F. The Findings on Bone Scintigraphy in Patients with Suspected Tumor-Induced Osteomalacia Should Not Be Overlooked. Clinical Nuclear Medicine. 2018;43(4):239EP-45. |
| 38 | Ward 2004 | Ward LM, Rauch F, White KE, Filler G, Matzinger MA, Letts M, et al. Resolution of severe, adolescent-onset hypophosphatemic rickets following resection of an FGF-23-producing tumour of the distal ulna. Bone. 2004;34(5):905EP-11. |
| 39 | Wu 2019 | Wu H, Bui MM, Zhou L, Li D, Zhang H, Zhong D. Phosphaturic mesenchymal tumor with an admixture of epithelial and mesenchymal elements in the jaws: clinicopathological and immunohistochemical analysis of 22 cases with literature review. Modern Pathology. 2019;32(2):189EP-204. |
| 40 | Xie 2013 | Xie Y, Li HZ. Oncogenic osteomalacia caused by renal cell carcinoma. Journal of Clinical Endocrinology and Metabolism. 2013;98(12):4597EP-8. |
| 41 | Xu 2009 | Xu M, Zhang Q, Peng D. A distal tibial tumor accompanied by tumor-induced osteomalacia. Orthopaedic surgery. 2009;1(3):245EP-9. |

3. Age- and sex- specific pediatric reference serum phosphate values

|  | **Age** | **Female reference interval** | | | | | **Male reference interval** | | | | |
| --- | --- | --- | --- | --- | --- | --- | --- | --- | --- | --- | --- |
|  |  | **Female Lower** | **Female Upper** | **Female n** | **Female Lower CI** | **Female Upper CI** | **Male Lower** | **Male Upper** | **Male n** | **Male Lower CI** | **Female Upper CI** |
| **Phosphate, mg/dL** | 0–14 days | 5.6 | 10.5 | 204 | 5.4–5.9 | 10.2–10.7 | 5.6 | 10.5 | 204 | 5.4–5.9 | 10.2–10.7 |
|  | 15 days–<1 year | 4.8 | 8.4 | 144 | 4.2–5.0 | 8.1–8.6 | 4.8 | 8.4 | 144 | 4.2–5.0 | 8.1–8.6 |
|  | 1–<5 years | 4.3 | 6.8 | 184 | 4.0–4.5 | 6.5–7.4 | 4.3 | 6.8 | 184 | 4.0–4.5 | 6.5–7.4 |
|  | 5–<13 years | 4.1 | 5.9 | 352 | 4.1–4.2 | 5.9–6.0 | 4.1 | 5.9 | 352 | 4.1–4.2 | 5.9–6.0 |
|  | 13–<16 years | **3.2** | **5.5** | **95** | **3.0–3.3** | **5.4–5.7** | **3.5** | **6.2** | **95** | **3.4–3.6** | **6.0–6.3** |
|  | 16–<19 years | 2.9 | 5.0 | 187 | 2.7–3.1 | 4.9–5.6 | 2.9 | 5.0 | 187 | 2.7–3.1 | 4.9–5.6 |

Source: Adapted from Colantonio et al 2012 (1).
Bolded text indicates sex-specific differences within the age partitions.

References

1.	Colantonio DA, Kyriakopoulou L, Chan MK, Daly CH, Brinc D, Venner AA, et al. Closing the gaps in pediatric laboratory reference intervals: a CALIPER database of 40 biochemical markers in a healthy and multiethnic population of children. Clin Chem. 2012;58(5):854–68.
